# Chinese Herbal Medicine as an Adjunctive Therapy Ameliorated the Incidence of Chronic Hepatitis in Patients with Breast Cancer: A Nationwide Population-Based Cohort Study

**DOI:** 10.1155/2017/1052976

**Published:** 2017-10-30

**Authors:** Kuo-Chin Huang, Hung-Rong Yen, Jen-Huai Chiang, Yuan-Chih Su, Mao-Feng Sun, Hen-Hong Chang, Sheng-Teng Huang

**Affiliations:** ^1^Department of Chinese Medicine, China Medical University Hospital, Taichung, Taiwan; ^2^School of Chinese Medicine, China Medical University, Taichung, Taiwan; ^3^Research Center for Traditional Chinese Medicine, Department of Medical Research, China Medical University Hospital, Taichung, Taiwan; ^4^Management Office for Health Data, China Medical University Hospital, Taichung, Taiwan; ^5^Graduate Institute of Integrated Medicine, School of Chinese Medicine, China Medical University, Taichung, Taiwan

## Abstract

We conducted a National Health Insurance Research Database-based Taiwanese nationwide population-based cohort study to evaluate whether Chinese herbal medicine (CHM) treatment decreased the incidence of chronic hepatitis in breast cancer patients receiving chemotherapy and/or radiotherapy. A total of 81171 patients were diagnosed with breast cancer within the defined study period. After randomly equal matching, data from 13856 patients were analyzed. Hazard ratios of incidence rate of chronic hepatitis were used to determine the influence and therapeutic potential of CHM in patients with breast cancer. The patients with breast cancer receiving CHM treatment exhibited a significantly decreased incidence rate of chronic hepatitis even across the stratification of age, CCI score, and treatments. The cumulative incidence of chronic hepatitis for a period of seven years after initial breast cancer diagnosis was also reduced in the patients receiving CHM treatment. The ten most commonly used single herbs and formulas were effective in protecting liver function in patients with breast cancer, where* Hedyotis diffusa* and Jia-Wei-Xiao-Yao-San were the most commonly used herbal agents. In conclusion, our study provided information that western medicine therapy combined with CHM as an adjuvant modality may have a significant impact on liver protection in patients with breast cancer.

## 1. Introduction

Breast cancer is the most common and the second most life-threatening cancer in women. Conventional breast cancer treatments include surgery, radiation, and medication. Medical therapies (chemotherapy, hormone therapy, and target therapy) are adopted to improve the disease-free ratio, overall survival ratio, and patients' quality of life. However, several adverse effects induced by medical treatments in patients with breast cancer include fatigue, phlebitis, alopecia, nausea, vomiting, mucositis, myelosuppression, cardiac toxicity, renal toxicity, and hepatotoxicity. Those adverse effects may affect up to 60% of patients and limit the application and efficacy of medical therapy [1]. Thus, resolving adverse effects is an important issue in clinical practice.

Complementary and alternative medicines (CAM) are widely applied to healthcare approaches throughout the world. A recent study showed that up to 87% of women with breast cancer reported CAM use [2]. Traditional Chinese medicine (TCM), a holistic system of medicine which includes herbs, acupuncture, food therapy, acupressure, massage, and therapeutic exercise that have been practiced for more than 3,000 years in China, is the most common CAM adopted for disease prevention and treatment in Taiwan. Patients with breast cancer frequently use CHM treatment to improve the adverse effects resulting from western medication. Several studies have demonstrated the modest efficacy of TCM in treating fatigue, nausea, vomiting, and myelosuppression. However, the extent and range of such evidence are still limited [1].

Hepatotoxicity is also a major side effect that may interrupt medical therapy of breast cancer. Patients with viral hepatitis frequently receive TCM for the drug resistance and dose-dependent side effects of antiviral agents. Recently, several studies have shown the efficacy of TCM to improve viral hepatitis [3, 4] and have also reported on the active compounds in several Chinese Herbal formulas or single herbs to discuss their possible mechanisms in viral hepatitis treatment [5]. However, only one study reported that TCM combined with chemotherapy for advanced breast cancer patients might have some effects on reducing toxicity in the liver and kidneys, but differences were not statistically significant [1]. Based on TCM theory, practitioners prescribe herbal formulas for specific diseases, according to patients' individual syndromes, symptoms, and patterns, and usually revise the formula based on patient response. It is difficult to process a powerful randomized control trial to truly abide by TCM theory. CMH granules are supported by the National Health Insurance (NHI) in Taiwan, including single Chinese herbs and multiherbal Chinese formulas. All of these CMH granules covered by the NHI program are manufactured by Good Manufacturing Practice- (GMP-) certified pharmaceutical companies. The daily clinical practice use of CMH granules is recorded in the NHI database. Herein, we conducted a population-based retrospective cohort study from the NHI database to evaluate the cumulative incidence of chronic hepatitis between CHM users and nonusers in patients diagnosed with breast cancer.

## 2. Materials and Methods

### 2.1. Data Source

This study used reimbursement claim data from the Taiwan National Health Insurance Program. An NHI program was implemented in March 1995, in which 22.6 million individuals from a total population of 23.0 million in Taiwan were enrolled. Currently, 99.6% of Taiwanese residents are covered by NHI. The National Health Insurance Research Database (NHIRD) is composed of every medical record reimbursed by the NHI. The datasets of the study consist of registry for beneficiaries, ambulatory, and inpatient care claims and Registry for Catastrophic Illness from NHIRD. We used ambulatory and inpatient care records for cancer care linking with the Registry for Catastrophic Illness patients in the period of 2000 to 2010 to identify study subjects for follow-up until the end of 2011. Ambulatory care claims contain individual's gender and birthday, visit date, and codes for the International Classification of Disease, Ninth Revision, and Clinical Modification (ICD-9-CM) for three primary diagnoses. Inpatient claims contain ICD-9-CM codes for principal diagnosis up to four secondary diagnoses. Registry for Catastrophic Illness database contains data from those insured who suffer from major diseases and are granted exemption from copayment. The ICD-9-CM codes were used for diagnosis by Chinese medicine physicians. Because the NHIRD contains identified secondary data for research, the present study was waived from informed consent. This study was approved by the Institutional Review Board of China Medical University Hospital (CMUH104-REC2-115).

### 2.2. Study Population

**Figure 1:**
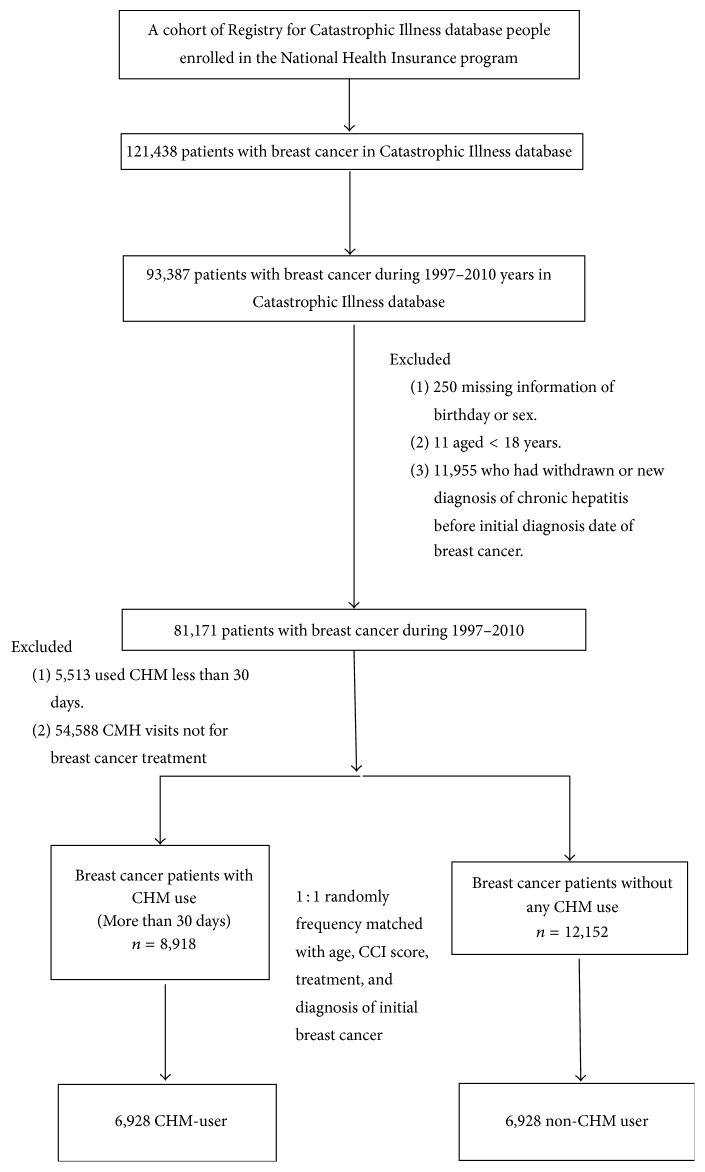
Study population flowchart diagram. Of the total amount of breast cancer patients registered in the NHIRD (*n* = 121438), 93387 patients with breast cancer were diagnosed within the years 1997–2010. After excluding patients with missing information as well as matching 1 : 1 by age, CCI score, treatment, and diagnosis of initial breast cancer, both groups contained 6928 patients.

A retrospective study was conducted using the Catastrophic Illness database from 1997 to 2011 years. Breast cancer patients (aged ≧18 years) who were diagnosed with ICD-9-CM code 174 were identified from the Catastrophic Illness database covering 1997 to 2010 and were followed up until December 31, 2011. In this study, we excluded patients receiving acupuncture or moxibustion and a diagnosis date of chronic hepatitis before breast cancer (Figure 1).

### 2.3. Primary Outcome

The primary outcome was chronic hepatitis (ICD-9-CM: 571.4) during the 14 years of follow-up. All eligible patients were followed up from the index date to December 31, 2011.

### 2.4. Exposure to Chinese Herbal Medicine

Patients using CHM for more than 30 days due to a diagnosis of breast cancer were defined as CHM users, whereas those without CHM outpatient records were defined as non-CHM users.

### 2.5. CCI Score and Treatments

The Charlson Comorbidity Index (CCI) score was used to determine patients' overall systematic health. The patients with treatments of radiotherapy and/or chemotherapy performed after the half year before diagnosis date of breast cancer were included.

### 2.6. Statistical Analysis

Continuous variables were reported as mean and standard deviation, whereas categorical variables were reported as number and percentage. Differences in proportions and means were evaluated by chi-square test or *t*-test. A Cox proportional hazard model accounting for age, CCI score, and treatment modality in the results, with a 95% confidence interval (CI), was used to estimate the hazard ratios (HR). For categorical covariates, Kaplan–Meier and log-rank tests were performed for the cumulative incidence of chronic hepatitis. A *p* value < 0.05 was considered statistically significant. In this study SAS 9.4 (SAS Institute Inc., Cary, NC) was used for statistical analysis.

## 3. Results

Of the 8918 CHM users and 12152 non-CHM users diagnosed with breast cancer from 1997 to 2010, after frequency matching both groups for age (per 5 years), CCI score, treatments, and initial diagnosis year of breast cancer, each group contained 6,928 patients. Cohort group and compared cohort group demonstrated similar characteristics without statistically significant differences (*p* > 0.05) (Table 1).

**Figure 2:**
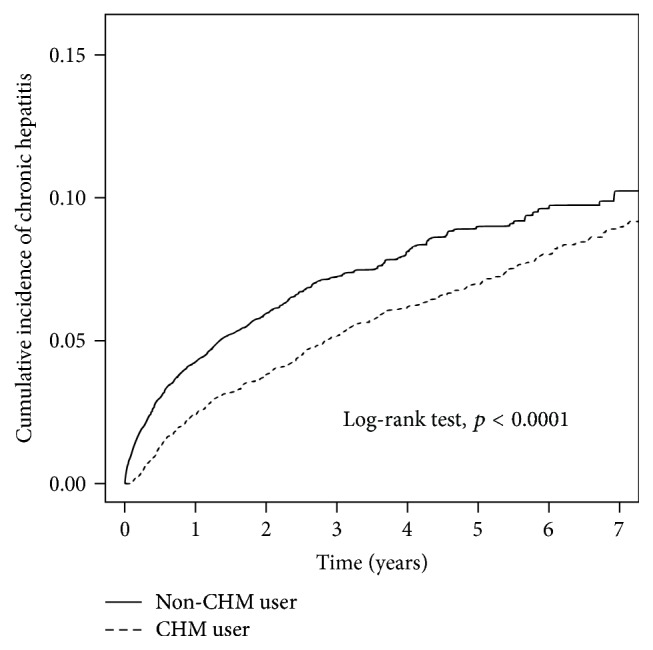
The estimated cumulative incidence of chronic hepatitis of those treated with CHM or none in the patients with breast cancer cohort by Kaplan–Meier analysis.

The difference in cumulative incidence of chronic hepatitis between the two groups was illustrated through a Kaplan–Meier analysis (Figure 2). The log-rank test revealed a significantly lower cumulative incidence of chronic hepatitis in the CHM cohort than that of the non-CHM cohort group (*p* < 0.0001). With or without adjusted HR by Cox proportional analysis, CHM users had lower risk of chronic hepatitis in comparison with non-CHM users (Table 2).

Stratified by age group, the incidence rates of chronic hepatitis in the 18–39-year group and 40–59-year group which used CHM were 13.12 and 15.17 per 1,000 person-years, respectively, which was lower than those in the comparison cohort (20.96 and 23.94 per 1,000 person-years). In addition, the 18–39-year group showed 0.65-fold (95% CI: 0.45–0.93) and the 40–59-year group showed 0.63-fold (95% CI: 0.53–0.73) lower risk of development of chronic hepatitis than the non-CHM cohort. As for the CCI score stratification, our results demonstrated that patients in the CHM-used cohort had lower risk and incidence rate of chronic hepatitis in comparison with the nonused CHM cohort group. There was statistical significance between the CHM users and non-CHM users in the subgroup of CCI score 0. We found that the CHM users also had the lower tendency to develop chronic hepatitis than those of non-CHM users; however, this lacks statistical significance as a result of small sample size of the other two subgroups (CCI scores 1 and ≥2) (Table 2). In individuals' accepted and nonaccepted treatment with radiotherapy or chemotherapy, patients in the CHM-used cohort had lower risk of chronic hepatitis compared to the non-CHM cohort group, with statistical significance (Table 2).

In Table 3, the 10 single herbs and multiherbal products (formulas) in TCM most prescribed for the treatment of patients with breast cancer are listed. The average daily dose of each single herb and formula is from 0.6 to 2 gram and from 4.1 to 6.6 grams, respectively. The duration for prescription of each single herb and formula is from 11.6 to 13.3 days and from 11.8 to 14.2 grams, respectively. The effects of TCM use were further explored using a Cox proportional hazard regression analysis. The results demonstrated all of these ten single herbs and formulas significantly decreased chronic hepatitis risk in patients with breast cancer, as shown in Table 4.

## 4. Discussion

The occurrence of liver injuries is often an impediment in clinical practice. Patients suffering from abnormal liver function during chemotherapy and/or radiotherapy may need to change the medication, modify the original dose, and even delay or terminate the treatment. Thus, the side effects induced by chemotherapy and/or radiotherapy may diminish the efficacy of treatment and lead to a drop in remission rate associated with failure to shrink tumor size, earlier relapse, and metastasis. Our study found that CHM treatment significantly decreased the incidence of chronic hepatitis after adjustment for age, CCI score, treatment modality, and medication used in patients with breast cancer. The cumulative incidence rates of chronic hepatitis in patients with breast cancer were also lower in CMH users in comparison with those of non-CMH users, up to the 7-year period. These findings indicate that CMH may be effective in protecting patients with breast cancer from liver injury.

As we know, the liver needs to confront the impact of drug toxicity and control major metabolism of xenobiotics taken from the gastrointestinal tract and portal circulation. There are several kinds of pathological injuries induced by drug-related hepatotoxicity, such as tissue necrosis, fibrosis, steatosis, steatohepatitis, cholestasis, and hepatic sinusoidal injury. The different modalities of treatment, including chemotherapy, hormone therapy, and targeted therapy frequently adopted in patients with breast cancer may have the possibility of inducing the side effect of hepatotoxicity. Medications such as fluorouracil, gemcitabine, capecitabine, cyclophosphamide, tamoxifen, letrozole, and trastuzumab might contribute to abnormal liver function independently or when combined and applied in clinical practice. In addition, liver injuries may also be exacerbated by other agents, including prophylactic antibiotics, antiemetics, and analgesic agents. Moreover, patients' previous medical illness, nutritional status, or chronic infections could also influence liver function. For example, Taiwan has a high prevalence of^,^ viral B and C hepatitis [6]. Breast cancer patients with a history as hepatitis virus carriers might suffer flare-up during the course of chemotherapy due to the immune-compromising result of myelosuppression or high-dose steroid for the management of nausea, vomiting, or poor appetite. Therefore, preventing liver injuries in patients with breast cancer is an important issue in clinical practice. With chemotherapy-associated hepatotoxicity, it is often difficult to detect clear pathogens and explore distinct mechanisms [7], which leads to complications in identifying specific treatment for protection. Thus, it is valuable to apply CHM to the prevention of hepatic injuries for breast cancer patients undergoing chemotherapy and/or radiotherapy.

CMH prescription is based on the holism of TCM pattern differentiation. According to the theory of TCM, the major cause of breast cancer is qi stagnation and blood stasis and the major associated meridians involved include the liver, kidneys, stomach, spleen, pericardium meridian, and gallbladder. Several herbs and herbal formulas adopted for moving qi perform functions such as promoting blood, clearing heat, resolving phlegm, and dissipating binds. Our study found Jia-Wei-Xiao-Yao-San (JWXYS), a popular herbal formula for liver stagnation and spleen deficiency, is the most frequently prescribed herbal formula for patients with breast cancer associated with hormone therapy [8]. In addition, San-Zhong-Kui-Jian-Tang (SZKJT) has been reported as effective in inhibiting breast cancer cell proliferation by induction of p21/WAF1 and activity of the mitochondrial apoptotic system [9]. Xue-Fu-Zhu-Yu-Tang (XFZYT) is also adopted in the treatment of prostate cancer, combined with western medication, based on the theory of blood stasis [10]. The most common single herb used to treat breast cancer in this study was* Hedyotis diffusa* (HD), which has been reported to exert an antiproliferative effect on breast cancer cells through apoptosis [11].* Taraxacum officinale* (TO), also known as dandelions, has been reported to be effective in suppressing breast cancer cell growth through extracellular signal-regulated kinase activities in vitro [12].* Scutellaria barbata* (SB) is also commonly used to treat various types of cancer. The molecular mechanism of SB is selective cytotoxicity, by inducting oxidative stress toward breast cancer cells [13].* Spatholobus suberectus* (SS) and* Salvia miltiorrhiza* (SM) are also prescribed for cancer patients because they are believed to be efficient in improving blood stasis. The possible inhibitory effects on breast cancer may be due to the induction of cell cycle arrest and apoptosis [14]. Epigallocatechin, a key compound of SS, is reported to inhibit the lactate dehydrogenase A in breast cancer [15]. The extracts of SM, including tanshinones I and IIa, salvianolic acid A, cryptotanshinone, isocryptotanshinone, and neo-tanshinlactone, reportedly inhibit breast cancer cell proliferation through cell growth arrest [16].

Some other formulas are adopted for resolving the side effects or complications resulting from western treatments of breast cancer. Sheng-Mai-Yin (SMY) is widely used to prevent excessive sweating and fatigue.* Panax ginseng*, one of major components in SMY, is reported to have an antifatigue effect in animal models [17]. Xiang-Sha-Liu-Jun-Zi-Tang (XSLJZT) is used to relieve gastrointestinal symptoms in patients receiving chemotherapy [8]. Bu-Zhong-Yi-Qi-Tang (BZYQT) is beneficial in treating cancer-related fatigue and improving general quality of life in cancer patients. A recent report indicated that BZYQT may be an adjunct used to enhance cisplatin-induced cancer cell apoptosis [18].* Astragalus membranaceus* (AM) is reported to inhibit breast cancer cell proliferation [19]. Isoflavones isolated from AM including formononetin, calycosin, and biochanin A have shown reversal effects in breast cancer cell proliferation. Formononetin and high concentrations of biochanin A have exhibited inhibitory effects on human breast cancer cells, while calycosin and low doses of biochanin A have exhibited stimulatory effects on the proliferation of breast cells through an estrogen receptor dependent mechanism [20]. Suan-Zao-Ren-Tang (SZRT) and Gui-Pi-Tang (GPT), which contained* Zizyphi Spinosi Semen* (ZSS) in their formula, are used to treat sleep disorders and major depressive disorders.* Polygonum multiflorum* Thunb. (PM) has demonstrated inhibitory effects on breast cancer cell proliferation by modulating the protein expression in cell cycle arrest and apoptosis [21]. Zhen-Ren-Huo-Ming-Yin (ZRHMY) is used for swollen sore and chronic hepatitis due to its anti-inflammatory effects and is applied in clinical practice for resolving complications or side effects induced by chemotherapy or radiotherapy [22].

In our study, the top 10 frequently prescribed herbal formulas and single herbs presented significant benefits in ameliorating incidence of chronic hepatitis. Several herbal formulas and single herbs adopted in breast cancer treatment were also frequently used in hepatitis treatment and provided protection from liver injury. JWXYS was the second most prescribed herbal formula for patients with chronic hepatitis in Taiwan [23]. It was reported to have the effects of decreasing transaminase, increasing serum albumin, and preventing liver fibrosis. The possible mechanism that protected the liver from chronic injury was considered to be through the stimulation of antioxidative activity [24]. A population-based cohort study revealed that the use of JYXYS may lower the mortality rate of chronic hepatitis B in patients receiving lamivudine treatment [25].* Schizandrae fructus*, one of the components of SMY, was reported to have protective effects in viral hepatitis or chemical-induced hepatic injury [26]. Additionally, many ingredients or extracts from CHM demonstrated protective effects from hepatotoxicity by HBV and/or HCV through antioxidative and anti-inflammatory pathways [27, 28]. Presently, there is insufficient research into the application of herbal formulas and herbs for liver protection in patients with breast cancer. This is the first report to present that CHM might effectively protect the liver from injury induced by chemotherapy or radiotherapy in patients with breast cancer.

This study obtained the data from the NHIRD, a government-run, single-payer NHI program that covers over 99% of the Taiwanese population and 93% of healthcare institutes [29], which ensures this study represents the general population with minimal selection bias and offers a comprehensive result of both CHM and non-CHM users among breast cancer patients. We could compare the long-term treatment effects by noting the incidence of subsequent chronic hepatitis. However, we did not have access to patients' exact physical examinations, laboratory data (transaminase, bilirubin, alkaline phosphatase, Gamma-glutamyl transferase, serum albumin, etc.), or lifestyle details, which were not collected in the NHI database. For this reason, we did not have patients' exact clinical condition to compare the severity of hepatitis. We also could not clarify if CHM users tend to maintain healthier lifestyle modifications, such as avoiding exposure to obesity or alcohol consumption, which could influence the incidence of hepatitis. Nevertheless, this population-based large-scale retrospective cohort analysis of the NHI database provides valuable information on CHM combination therapy in breast cancer patients with chemotherapy and/or radiotherapy treatment.

## 5. Conclusion

This population-based, retrospective cohort study showed that CHM combination therapy might decrease the risk of chronic hepatitis in breast cancer patients with chemotherapy and/or radiotherapy treatment. Our study results suggested CHM as an adjunctive therapy in breast cancer patients receiving chemotherapy and/or radiotherapy to prevent subsequent chronic hepatitis. Future clinical study is needed to substantiate the relationship of CHM to reduced hepatotoxicity in patients with breast cancer receiving chemotherapy and/or radiotherapy.

## Supplementary Material

Supplementary Table 1. The composition of ten formulas. Supplementary Table 2. The biological activities associated with experimental models of extracted ingredients in herbal formulas and single herbs.

## Figures and Tables

**Table 1:** Characteristics of breast cancer patients according to use and nonuse of Chinese herb.

Variable	Breast cancer patients				*p* value
	Chinese herb used				
	No (*N* = 6928)		Yes (*N* = 6928)		
	*n*	%	*n*	%	
*Age group*					0.99
18–39	1056	15.24	1056	15.24	
40–59	5377	77.61	5377	77.61	
≥60	495	7.14	495	7.14	
Mean ± SD (years)^a^	49.59 (9.73)		49.56 (9.73)		0.8371
*Baseline CCI score *					0.99
0	6491	93.69	6491	93.69	
1	227	3.28	227	3.28	
≥2	210	3.03	210	3.03	
*Treatment*					
Radiotherapy					0.99
No	3137	45.28	3137	45.28	
Yes	3791	54.72	3791	54.72	
Chemotherapy					0.99
No	1291	18.63	1291	18.63	
Yes	5637	81.37	5637	81.37	
*Drug used*					
Fluorouracil	1275	18.4	2166	31.26	<0.0001
Gemcitabine	353	5.1	562	8.11	<0.0001
Capecitabine	601	8.67	804	11.61	<0.0001
Cyclophosphamide	1336	19.28	2466	35.59	<0.0001
Tamoxifen	3105	44.82	3842	55.46	<0.0001
Letrozole	855	12.34	1155	16.67	<0.0001
Trastuzumab	483	6.97	594	8.57	0.0004
*Follow time (mean, median)*	2.73 (1.94)		4.32 (3.55)		

Chi-square test;^a^  *t*-test.

**Table 2:** Incidence rates, hazard ratio, and confidence intervals of chronic hepatitis for breast cancer patients with and without Chinses herb used in the stratification of sex, age, CCI score, and treatment.

Variables	Chinese herb used						Crude HR	Adjusted HR
	No			Yes				
	(*N* = 6928)			(*N* = 6928)				
	Event	Person-years	IR^†^	Event	Person-years	IR^†^	(95% CI)	(95% CI)
*Total*	442	18931	23.35	441	29910	14.74	0.74 (0.64–0.84)^*∗∗∗*^	0.63 (0.54–0.72)^*∗∗∗*^
*Age*								
18–39	66	3149	20.96	65	4955	13.12	0.69 (0.49–0.98)^*∗*^	0.65 (0.45–0.93)^*∗*^
40–59	351	14662	23.94	352	23198	15.17	0.75 (0.64–0.87)^*∗∗∗*^	0.63 (0.53–0.73)^*∗∗∗*^
≥60	25	1119	22.34	24	1757	13.66	0.71 (0.4–1.25)	0.64 (0.36–1.15)
*CCI score*								
0	424	17983	23.58	424	28321	14.97	0.74 (0.65–0.85)^*∗∗∗*^	0.62 (0.54–0.72)^*∗∗∗*^
1	11	486	22.64	13	858	15.15	0.68 (0.3–1.57)	0.66 (0.28–1.54)
≥2	7	462	15.16	4	731	5.47	0.44 (0.13–1.5)	0.47 (0.13–1.69)
*Treatment*								
Radiotherapy								
No	263	9275	28.35	253	14587	17.34	0.72 (0.61–0.86)^*∗∗∗*^	0.58 (0.48–0.69)^*∗∗∗*^
Yes	179	9655	18.54	188	15323	12.27	0.75 (0.61–0.92)^*∗∗*^	0.7 (0.56–0.87)^*∗∗*^
Chemotherapy								
No	89	3842	23.17	85	5780	14.71	0.74 (0.55–0.99)^*∗*^	0.68 (0.5–0.93)^*∗*^
Yes	353	15089	23.39	356	24130	14.75	0.73 (0.63–0.85)^*∗∗∗*^	0.61 (0.52–0.72)^*∗∗∗*^

Crude HR^*∗*^ represented relative hazard ratio; adjusted HR^†^ represented adjusted hazard ratio: mutually adjusted for Chinese herb used, age, CCI score, treatment, and lag time in Cox proportional hazard regression. Lag time was defined as the duration between first diagnosis breast cancer dates and first accepted Chinese herb medicine date during the follow-up period. ^*∗*^*p* < 0.05, ^*∗∗*^*p* < 0.01, and ^*∗∗∗*^*p* < 0.001.

**Table 3:** Ten most common herbs and formulas prescribed.

Herbal formula	Frequency	Number of person-days	Average daily dose	Average duration for prescription
			(g)	(Days)
*Single herb*				
*Hedyotis diffusa*	28428	368769	1.5	13
*Taraxacum officinale*	20713	260518	1.7	12.6
*Scutellaria barbata*	19329	249317	2	12.9
*Spatholobus suberectus*	12432	163265	1.7	13.1
*Salvia miltiorrhiza*	11319	137929	1.6	12.2
Zizyphi Spinosi Semen	9981	124473	1.9	12.5
*Astragalus membranaceus*	10585	122984	1.7	11.6
Rhei Rhizoma	9590	111498	0.6	11.6
Polygonum multiflorum Thunb.	8338	110657	1.4	13.3
*Fritillaria thunbergii* Miq.	8459	98822	1.5	11.7
*Formula*				
Jia-Wei-Xiao-Yao-San	23987	302757	5.3	12.6
Xiang-Sha-Liu-Jun-Zi-Tang	9450	123266	4.2	13
San-Zhong-Kui-Jian-Tang	9672	113868	4.6	11.8
Suan-Zao-Ren-Tang	6901	98059	4.1	14.2
Gui-Pi-Tang	7367	92932	5	12.6
Zhen-Ren-Huo-Ming-Yin	6797	88308	4.4	13
Bu-Zhong-Yi-Qi-Tang	7445	87686	4.6	11.8
Zhi-Bai-Di-Huang-Wan	6081	81750	4.5	13.4
Xue-Fu-Zhu-Yu-Tang	6022	78473	6.6	13
Sheng-Mai-Yin	6602	78261	4.3	11.9

**Table 4:** Hazard ratios and 95% confidence intervals of chronic hepatitis risk associated with Chinese herbal formulas used among breast cancer patients.

CHM prescription	Chronic hepatitis		Hazard ratio (95% CI)	
	*N*	Number of events	Crude^*∗*^	Adjusted^†^
*Non-TCM user*	6928	442	1 (reference)	1 (reference)
*CHM user: single herb*				
*Hedyotis diffusa*	1809	104	0.68 (0.55–0.84)^*∗∗∗*^	0.51 (0.41–0.63)^*∗∗∗*^
*Taraxacum officinale*	2161	117	0.64 (0.52–0.78)^*∗∗∗*^	0.46 (0.37–0.57)^*∗∗∗*^
*Scutellaria barbata*	1416	81	0.64 (0.50–0.81)^*∗∗∗*^	0.47 (0.37–0.60)^*∗∗∗*^
*Spatholobus suberectus*	1640	91	0.66 (0.53–0.83)^*∗∗∗*^	0.48 (0.38–0.61)^*∗∗∗*^
*Salvia miltiorrhiza*	1706	110	0.71 (0.58–0.88)^*∗∗*^	0.53 (0.43–0.65)^*∗∗∗*^
Zizyphi Spinosi Semen	1578	84	0.60 (0.47–0.76)^*∗∗∗*^	0.45 (0.35–0.57)^*∗∗∗*^
*Astragalus membranaceus*	1716	111	0.74 (0.60–0.91)^*∗∗*^	0.54 (0.44–0.67)^*∗∗∗*^
Rhei Rhizoma	957	36	0.44 (0.32–0.62)^*∗∗∗*^	0.33 (0.23–0.46)^*∗∗∗*^
Polygonum multiflorum Thunb.	1383	81	0.68 (0.54–0.86)^*∗∗*^	0.53 (0.41–0.67)^*∗∗∗*^
*Fritillaria thunbergii* Miq.	1467	67	0.52 (0.40–0.68)^*∗∗∗*^	0.40 (0.31–0.52)^*∗∗∗*^
*CHM user: formula*				
Jia-Wei-Xiao-Yao-San	2814	180	0.69 (0.58–0.83)^*∗∗∗*^	0.55 (0.46–0.65)^*∗∗∗*^
Xiang-Sha-Liu-Jun-Zi-Tang	1549	98	0.73 (0.58–0.91)^*∗∗*^	0.54 (0.43–0.68)^*∗∗∗*^
San-Zhong-Kui-Jian-Tang	879	47	0.66 (0.49–0.90)^*∗∗*^	0.50 (0.37–0.67)^*∗∗∗*^
Suan-Zao-Ren-Tang	1253	69	0.62 (0.48–0.80)^*∗∗∗*^	0.48 (0.37–0.62)^*∗∗∗*^
Gui-Pi-Tang	1369	76	0.65 (0.51–0.83)^*∗∗∗*^	0.49 (0.38–0.63)^*∗∗∗*^
Zhen-Ren-Huo-Ming-Yin	935	36	0.45 (0.32–0.63)^*∗∗∗*^	0.34 (0.24–0.47)^*∗∗∗*^
Bu-Zhong-Yi-Qi-Tang	1356	84	0.69 (0.55–0.88)^*∗∗*^	0.53 (0.42–0.67)^*∗∗∗*^
Zhi-Bai-Di-Huang-Wan	1142	69	0.69 (0.53–0.88)^*∗∗*^	0.52 (0.40–0.67)^*∗∗∗*^
Xue-Fu-Zhu-Yu-Tang	1101	56	0.58 (0.44–0.76)^*∗∗∗*^	0.45 (0.34–0.59)^*∗∗∗*^
Sheng-Mai-Yin	1147	61	0.63 (0.48–0.82)^*∗∗∗*^	0.47 (0.36–0.62)^*∗∗∗*^

Crude HR^*∗*^ represented relative hazard ratio; adjusted HR^†^ represented adjusted hazard ratio: mutually adjusted for Chinese herb used, age, CCI score, treatment, and lag time in Cox proportional hazard regression. Lag time was defined as the duration between first diagnosis breast cancer dates and first accepted Chinese herb medicine date during the follow-up period. ^*∗*^*p* < 0.05, ^*∗∗*^*p* < 0.01, and ^*∗∗∗*^*p* < 0.001.

## Abbreviations

AM: * Astragalus membranaceus*
BZYQT: Bu-Zhong-Yi-Qi-Tang
CAM: Complementary and alternative medicines
CCI: Charlson Comorbidity Index
CHM: Chinese herb medicine
CI: Confidence interval
GMP: Good Manufacturing Practice
GPT: Gui-Pi-Tang
HD: *Hedyotis diffusa*
HR: Hazard ratios
ICD-9-CM: International Classification of Disease, Ninth Revision, and Clinical Modification
JWXYS: Jia-Wei-Xiao-Yao-San
NHI: National Health Insurance
NHIRD: National Health Insurance Research Database
PM: *Polygonum multiflorum* Thunb.
SB: *Scutellaria barbata*
SM: *Salvia miltiorrhiza*
SMY: Sheng-Mai-Yin
SS: *Spatholobus suberectus*
SZKJT: San-Zhong-Kui-Jian-Tang
SZRT: Suan-Zao-Ren-Tang
TCM: Traditional Chinese medicine
TO: *Taraxacum officinale*
XFZYT: Xue-Fu-Zhu-Yu-Tang
XSLJZT: Xiang-Sha-Liu-Jun-Zi-Tang
ZSS: *Zizyphi Spinosi Semen*
ZRHMY: Zhen-Ren-Huo-Ming-Yin.
