# Supplementary material for: Chinese Herbal Medicine as an Adjunctive Therapy Ameliorated the Incidence of Chronic Hepatitis in Patients with Breast Cancer: A Nationwide Population-Based Cohort Study
Source: Evid Based Complement Alternat Med. 2017 Oct 30;2017:1052976. doi: 10.1155/2017/1052976 (PMC5682887; doi:10.1155/2017/1052976)
Supplement: Supplementary file 1 — Supplementary Table 1. The composition of ten formulas. Supplementary Table 2. The biological activities associated with experimental models of extracted ingredients in herbal formulas and single herbs. [file 1052976.f1.docx]

**Supplementary Table 1. The composition of ten formulas**

| **Ten formulas** | **Compositions** |
| --- | --- |
| Jia-Wei-Xiao-Yao-San | Angelica sinensis, *Wolfiporia extensa*, *Gardenia jasminoides*, *Mentha*, *Paeonia lactiflora*, Bupleurum chinense, *Glycyrrhiza uralensis*, *Atractylodis macrocephalae*, *Paeonia suffruticosa*, *Zingiber officinale* |
| Xiang-Sha-Liu-Jun-Zi-Tang | *Panax ginseng*, *Atractylodis macrocephalae*, *Wolfiporia extensa*, *Glycyrrhiza uralensis*, Pinellia ternata, *Citrus reticulata Blanco*, *Saussurea costus*, *Amomum xanthioides*, *Zingiber officinale* |
| San-Zhong-Kui-Jian-Tang | *Scutellaria baicalensis, Anemarrhena asphodeloides, Phellodendron chinense Schneid., Gentiana scabra, Trichosanthes kirilowii, Platycodon grandiflorus, Laminaria japonica, Bupleurum chinense, Cimicifuga foetida, Forsythia suspensa, Glycyrrhiza uralensis, Sparganium stoloniferum, Atractylodis macrocephalae, Pueraria montana, Angelica sinensis,* *Paeonia lactiflora, Coptis chinensis* |
| Suan-Zao-Ren-Tang | *Zizyphi Spinosi Semen, Glycyrrhiza uralensis, Anemarrhena asphodeloides, Wolfiporia extensa, Ligusticum wallichi* |
| Gui-Pi-Tang | *Panax ginseng*, *Astragalus membranaceus*, *Atractylodis macrocephalae*, *Wolfiporia extensa*, Angelica sinensis, *Zizyphi Spinosi Semen*, *Dimocarpus longan*, Polygala tenuifolia, *Saussurea costus*, *Glycyrrhiza uralensis*, *Zingiber officinale*, *Ziziphus jujube* |
| Zhen-Ren-Huo-Ming-Yin | *Lonicera japonica, Citrus reticulata Blanco, Angelica sinensis, Saposhnikovia divaricata, Angelica dahurica, Glycyrrhiza uralensis, Fritillaria thunbergii Miq., Trichosanthes kirilowii, Boswellia thurifera, Commiphora myrrha, Gleditsia sinensis* |
| Bu-Zhong-Yi-Qi-Tang | *Astragalus membranaceus*, *Glycyrrhiza uralensis*, *Panax ginseng*, *Angelica sinensis*, *Citrus reticulata Blanco*, *Cimicifuga foetida*, Bupleurum chinense, *Atractylodis macrocephalae* |
| Zhi-Bai-Di-Huang-Wan | *Rehmannia glutinosa, Cornus officinalis, Dioscorea opposite, Wolfiporia extensa, Paeonia suffruticosa, Alisma orientalis, Anemarrhena asphodeloides, Phellodendron chinense Schneid.,* |
| Xue-Fu-Zhu-Yu-Tang | *Angelica sinensis, Rehmannia glutinosa, Prunus persica, Carthamus tinctorius, Citrus aurantium, Paeonia lactiflora, Bupleurum chinense, Glycyrrhiza uralensis, Platycodon grandiflorus, Ligusticum wallichii, Cyathula officinalis* |
| Sheng-Mai-Yin | *Panax ginseng*, *Liriope spicata*, *Schisandra chinensis* |

**Supplementary Table 2. The biological activities associated with experimental models of extracted ingredients in herbal formulas and single herbs**

| **Herbal formulas/ single herbs** | **Form** | **Biological effects and related molecular mechanisms** | **Models** |
| --- | --- | --- | --- |
| Jia-Wei-Xiao-Yao-San | granules | decreasing transaminase, increasing serum albumin, and preventing liver fibrosis through inhibit reactive-oxygen-species induced membrane lipid peroxidation | Rat [24] |
| San-Zhong-Kui-Jian-Tang | water extract | inhibiting breast cancer cell proliferation by induction of p21/WAF1 and activity of the mitochondrial apoptotic system | Human breast cancer cell; nude mice [9] |
| Bu-Zhong-Yi-Qi-Tang | water extract | enhance cisplatin-induced cancer cell apoptosis by increase of p53 expression and active caspase 3 and decrease of Akt phosphorylation and Bcl‑2/Bax ratio | Human cervical cancer cell [18] |
| *Hedyotis diffusa* | ethanol extract; Pure compound (ursolic and oleanolic acids) | upregulate p53 expression through ERα/Sp1-Mediated pathway | Human breast cancer cell [11] |
| *Taraxacum officinale* | water extract | suppressing breast cancer cell growth through extracellular signal-regulated kinase dependent manner | Human breast cancer cell [12] |
|  | ethanol extract | against HBV by blocking protein synthesis steps and DNA replication | Duck fetal hepatocytes; human hepatoma cell [27] |
|  | ethanol extract | protective effects from hepatotoxicity by anti-oxidative and anti-inflammatory pathways | Rat [27] |
|  | methanol extract | blocked the viral replication and NS5B gene expression | Human hepatoma cell (Huh-7) [28] |
| *Scutellaria barbata* | water extract | induce oxidative stress by the changes in expression of redox potential maintaining enzymes; caused depletion of ATP and NAD(H) by by inhibiting the major energy-producing pathways that are preferentially activated in tumor cells | Breast cancer cells [13] |
| *Spatholobus suberectus* | water extract | induction of cell cycle arrest at G2/M phase and apoptosis by DNA damage and activation of phosphor-Chk1/Chk2 | Human breast cancer cell [14] |
|  | water extract | inhibit the lactate dehydrogenase A activities | Human breast cancer cell [15] |
|  | pure compound (Epigallocatechin) | inhibit the lactate dehydrogenase A activities | Human breast cancer cell; nude mice [15] |
| *Salvia miltiorrhiza* | ethanol extract | inhibit breast cancer cell proliferation by down-regulation of Akt phosphorylation and an increase in p27 | Human breast cancer cell [16] |
| *Astragalus membranaceus* | pure compound (astragaloside IV, formononetin) | inhibit breast cancer cell proliferation by down-regulation of Akt phosphorylation | Human breast cancer cell [19] |
| *Polygonum multiflorum Thunb* | ethanol extract | inducing cell cycle arrest in the G2/M phase and promoting cell apoptosis by down-regulated the protein expression of Cdc25B and Cdc25C phosphatases, increase in phospho-Cdk1, and promoted cytochrome c release from mitochondria into the cytosol to activate caspase-9. | Human breast cancer cell [21] |
